# Sympatric Occurrence of 3 Arenaviruses, Tanzania

**DOI:** 10.3201/eid1604.091721

**Published:** 2010-04

**Authors:** Joëlle Goüy de Bellocq, Benny Borremans, Abdul Katakweba, Rhodes Makundi, Stuart J.E. Baird, Beate Becker-Ziaja, Stephan Günther, Herwig Leirs

**Affiliations:** University of Antwerp, Antwerp, Belgium (J. Goüy de Bellocq, B. Borremans, H. Leirs); Sokoine University of Agriculture, Morogoro, Tanzania (A. Katakweba, R. Makundi); Research Center in Biodiversity and Genetic Resources, Vairão, Portugal (J. Goüy de Bellocq, S.J.E. Baird); Bernhard-Nocht-Institute for Tropical Medicine, Hamburg, Germany (B. Becker-Ziaja, S. Günther); University of Aarhus, Kongens Lyngby, Denmark (H. Leirs)

**Keywords:** Old World arenaviruses, Murinae, RT-PCR, Tanzania, viruses, dispatch

## Abstract

To determine the specificity of Morogoro virus for its reservoir host, we studied its host range and genetic diversity in Tanzania. We found that 2 rodent species other than *Mastomys natalensis* mice carry arenaviruses. Analysis of 340 nt of the viral RNA polymerase gene showed sympatric occurrence of 3 distinct arenaviruses.

Arenaviruses are RNA viruses, primarily rodent borne, that include the etiologic agents of lymphocytic choriomeningitis and hemorrhagic fevers in humans. On the basis of their antigenic properties, arenaviruses have been divided in 2 groups: New World and Old World (*1*). In Africa, 2 arenaviruses are known to be highly pathogenic to humans: Lassa virus in West Africa and the recently described Lujo virus from southern Africa (*2*). Rodents from the subfamily Murinae are the principal hosts of the Old World arenaviruses. The multimammate mouse, *Mastomys natalensis*, is the reservoir host of Lassa virus in western Africa (*3*) and Mopeia virus, for which human pathogenicity has not been reported, in eastern Africa (*4*,*5*).

Previously, a serosurvey of small mammals from Tanzania identified a hot spot of arenavirus circulation in Morogoro (*6*). Molecular screening detected a new arenavirus in *M. natalensis* mice: Morogoro virus, closely related to Mopeia virus (*6*). This virus seems a promising model for studying virus–host dynamics and testing rodent control measures for arenaviruses for which *M. natalensis* mice are host. However, before being used as a model, the degree of specificity of Morogoro virus for its reservoir host must be assessed because secondary reservoir species may play a role in the transmission and maintenance of the virus in natural habitats. Our objective, therefore, was to determine the limit of specificity of the Morogoro virus.

## The Study

From October 13 through December 3, 2008, a total of 555 small mammals were trapped in Morogoro, Tanzania (6.84°S, 37.65°E). This period corresponds to the end of the dry season, when the density *of M. natalensis* mice is usually high (*7*). Sherman traps were set in habitats where these mice were expected to occur in high density (*7*). Dried blood samples were preserved on calibrated, prepunched filter papers. Blood samples (1 punch ≈15µL/rodent) were eluted in 300 µL of phosphate-buffered saline and tested for antibodies to arenaviruses by indirect immunofluorescence antibody (IFA) testing using Morogoro virus as antigen. In addition, total RNA was extracted from another punch of blood by using 300 µL of AVL buffer (QIAGEN, Venlo, the Netherlands). The lysate, plus 300 µL of ethanol, was centrifuged in a silica column (Zymo Research, Orange, CA, USA). The column was washed with 400 µL of AW1 and AW2 buffers (QIAGEN). RNA was eluted with 15 µL of water. A 1-step reverse transcription–PCR (RT-PCR) selective for the Morogoro virus RNA polymerase (large [L]) gene was performed as described (*6*); it was based on a pan–Old World arenavirus RT-PCR approach (*8*), but primers were adjusted to detect the Morogoro virus (*6*). Products were shown by agarose gel electrophoresis. A subset of the amplicons was purified and unidirectionally sequenced by using MoroL3359-fwd primer (*6*). Amplicons derived from *Lemniscomys rosalia* and *Mus minutoides* mouse blood samples were bidirectionally sequenced. Nucleotide and amino acid sequences were aligned by using BioEdit software (*9*). Old World arenaviruses and 2 representatives of New World arenaviruses were used to estimate nucleotide and amino acid pairwise divergence (p-distance) with MEGA 4 (*10*). A phylogram was reconstructed by using the neighbor-joining algorithm in MEGA 4.

We trapped 511 *M. natalensis* mice and 44 individuals from 7 other small mammal species (Table 1). IFA results were positive for 58 blood samples from *M. natalensis*, 1 from *L. rosalia*, and 1 from *M. minutoides* mice (Table 1). *M. rosalia* mice were trapped in woodlands, whereas *M. minutoides* mice were trapped in vegetable gardens and fallow fields. These results are consistent with recently reported results from a study in the same locality 20 years ago, in which mice from the genera *Lemniscomys* and *Mus* were seropositive for arenaviruses according to IFA with Lassa virus as antigen (*6*). In our 2008 study, the antibody prevalence for *M. natalensis* mice was 12.1%, which is low compared with 50% antibody prevalence reported for 2004 and 2007 (*6*), suggesting high fluctuation of interannual or seasonal prevalence of Morogoro virus in its host.

**Table 1:** Arenavirus antibodies and arenaviruses in blood samples of small mammals around Morogoro, Tanzania, October 13–December 3, 2008

Species	No. trapped	Antibodies*			Arenaviruses†		Total positive, no. (%)
		No. examined	No. (%) positive		No. examined	No. (%) positive	
*Acomys spinosissimus*	1	1	0		1	0	0
*Crocidura sp.*	20	11	0		12	0	0
*Dasymys incomtus*	1	1	0		1	0	0
*Lemniscomys rosalia*‡	3	3	1 (33.3)		3	1 (33.3)	2 (66)
*Mastomys natalensis*	511	480	58 (12.1)		489	41 (8.4)	93 (19)
*Mus minutoides*‡	7	5	1 (20)		5	1 (20)	2 (40)
*Rattus rattus*	1	1	0		1	0	0
*Gerbilliscus robustus*	11	11	0		11	0	0
Total	555	513	60 (11.7)		523	43 (8.2)	97 (18.5)

*Detected by indirect immunofluorescent antibody testing. †Large segment. Detected by reverse transcription–PCR. ‡Species identification was confirmed by sequencing mitochondrial cytochrome b gene.

Using RT-PCR selective for the arenavirus L gene, we obtained positive results for 43 mice: 41 *M. natalensis*, 1 *L. rosalia*, and 1 *M. minutoides*. In total, 6 samples were positive according to IFA and RT-PCR. We sequenced 33 RT-PCR amplicons. The sequences derived from *M. natalensis* mice (286 bp used for the analysis) showed 97.1%–100% amino acid homology with the Morogoro prototype L sequence (GenBank accession no. EU914104). In contrast, the 2 sequences derived from the blood samples of *L. rosalia* and *M. minutoides* mice showed only 69.3% and 65.2% aa homology with the Morogoro prototype L sequence. These sequences (320 bp) were compared with sequences of the Old World arenaviruses (Table 2). The virus amino acid sequence from *M. minutoides* mice clustered at 93.7% homology with that of the Kodoko virus, in the lymphocytic choriomeningitis clade (Figure; Table 2): thus, the arenavirus of *M. minutoides* mice seems to be a strain of the Kodoko virus originally isolated from 2 *M. minutoides* mice in Guinea (*11*). Our finding supports *M. minutoides* mice as the true reservoir of Kodoko virus in Africa.

**Table 2:** Nucleotide and amino acid p-distances of 2 arenaviruses in blood of *Mus minutoides* and *Lemniscomys rosalia* mice in Morogoro, Tanzania, October 13–December 3, 2008, compared with Old World and 2 New World arenaviruses*

Virus sequence†	Old World arenaviruses												New World arenaviruses	
	Dandedong	Ippy	Kodoko	Mobala	Mopeia	Morogoro	Lassa	LCMV	Lujo	Lemn	Minu		Pirital	Pichinde
Dandedong		0.282	0.073	0.218	0.255	0.282	0.209	0.027	0.355	**0.245**	**0.091**		0.418	0.409
Ippy	0.345		0.264	0.227	0.209	0.209	0.191	0.300	0.345	**0.173**	**0.273**		0.491	0.482
Kodoko	0.244	0.354		0.200	0.227	0.255	0.227	0.091	0.336	**0.236**	**0.073**		0.409	0.409
Mobala	0.318	0.315	0.315		0.136	0.127	0.145	0.227	0.327	**0.209**	**0.236**		0.482	0.464
Mopeia	0.310	0.295	0.351	0.256		0.055	0.145	0.273	0.345	**0.182**	**0.255**		0.482	0.464
Morogoro	0.327	0.324	0.360	0.262	0.241		0.136	0.282	0.364	**0.209**	**0.282**		0.491	0.473
Lassa	0.286	0.283	0.366	0.286	0.271	0.262		0.209	0.364	**0.200**	**0.236**		0.473	0.436
LCMV	0.182	0.354	0.223	0.339	0.324	0.345	0.330		0.373	**0.264**	**0.109**		0.436	0.409
Lujo	0.351	0.393	0.360	0.372	0.387	0.405	0.399	0.357		**0.373**	**0.336**		0.500	0.500
Lemn	**0.333**	**0.315**	**0.336**	**0.292**	**0.313**	**0.307**	**0.301**	**0.327**	**0.351**		**0.227**		0.482	0.464
Minu	**0.211**	**0.360**	**0.241**	**0.301**	**0.348**	**0.348**	**0.339**	**0.244**	**0.354**	**0.324**			0.436	0.409
Pirital	0.440	0.467	0.429	0.420	0.452	0.443	0.455	0.432	0.476	0.473	0.417			0.173
Pichinde	0.426	0.446	0.432	0.458	0.476	0.467	0.473	0.452	0.446	0.443	0.435		0.268	

*Nucleotides below diagonal; amino acids above diagonal. LCMV, lymphocytic choriomeningitis virus; Lemn, virus sequenced from *Lemniscomys rosalia* mice (indicated by **boldface**); Minu, virus sequenced from *Mus minutoides* mice (indicated by **boldface)*.*** †Strains and GenBank accession numbers of the sequences used: Dandenong (0710-2678, EU136039), Ippy (Dak An B 188 d, DQ328878), Kodoko (KD42, EF179865), Mobala (Acar 3080, DQ328876), Mopeia (Mozambique, DQ328875), Morogoro (3017/2004, EU914104), Lassa (Josiah, AY628202), LCMV (Armstrong,,AY847351), Lujo (NC_012777), Pirital (VAV 488, AY216505), Pichinde (AN3739, NC_006439). Sequences of arenaviruses in *L. rosalia* and *M. minutoides* mice have been deposited in GenBank under accession nos. GU182412 and GU182413, respectively.

The amino acid sequence of the virus isolated from *L. rosalia* mice clusters with the Ippy virus sequence (Figure). Ippy virus was isolated in the Central African Republic from *Arvicanthis niloticus* rodents (*12*). For the portion of L gene sequenced (320 bp), the level of amino acid divergence between the 2 is 17.3%, higher than the level of divergence between other Old World arenavirus species (e.g., 14.5% aa divergence between Mobala and Lassa viruses; Table 2). Thus, the arenavirus found in *L. rosalia* mice appears to be a new species of Old World arenavirus. The genus *Lemniscomys* is more closely related to the genus *Arvicanthis* than to the genera *Mus* and *Mastomys*.

**Figure:**
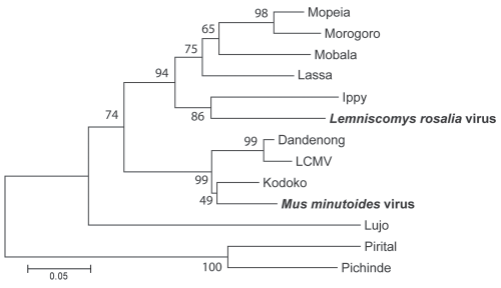
Neighbor-joining tree of Old World arenaviruses, showing position of 2 arenaviruses found in blood samples of *Lemniscomys rosalia* and *Mus minutoides* mice (**boldface**), based on the analysis of partial sequences of the RNA polymerase gene. Phylogeny was estimated by neighbor-joining of amino acid pairwise distance in MEGA 4 (*10*). Numbers represent percentage bootstrap support (1,000 replicates). Two New World arenaviruses, Pirital and Pichinde, were used as outgroups. See Table 2 for virus strains and GenBank accession numbers. Scale bar indicates amino acid substitutions per site. LCMV, lymphocytic choriomeningitis virus.

## Conclusions

In high-density habitats of *M. natalensis* mice, where Morogoro arenavirus transmission occurs, sympatric murine species do not seem to be secondary reservoirs for the virus. In contrast, 2 mouse species, *L. rosalia* and *M.* *minutoides*, seem to be reservoirs of 2 other Old World arenaviruses, 1 of which may be a new species. Our study emphasizes the complementary nature of serologic and genetic-based approaches for arenavirus detection. Because of the cross-reactivity of Morogoro antigens with immune serum from individuals infected with other arenaviruses, a serology-only approach might have led to the conclusion that an extended set of hosts exists for the Morogoro virus. Because of its high cost, a genetics-only approach might never have indicated the hot spot of arenavirus around Morogoro that was shown by IFA (*6*). However, critically, genetics then allow cross-reactivity to be decomposed.

Our study demonstrates the presence of 3 Old World arenaviruses in a single location. To date, only 5 Old World arenavirus species and 17 New World arenaviruses have been recognized by the International Committee for Taxonomy of Viruses (*13*). Although the likely presence of additional arenaviruses in Africa has long been suggested (*14*,*15*), the discovery of new Old World arenaviruses is rare. Our study illustrates that arenaviruses in Africa may be highly diverse and demonstrates the efficiency of the recently developed pan–Old World arenavirus RT-PCR for identifying new Old World arenaviruses (*8*). To isolate and describe the new arenavirus of *L.* *rosalia* mice and the strain of Kodoko virus, additional sampling and genotyping are being conducted. In particular, determining the sequence of the S segment will further clarify evolutionary relationships within the Old World group.
